# Effectiveness of biofeedback therapy on low anterior resection syndrome: a systemic review with meta-analysis

**DOI:** 10.3389/fmed.2025.1538114

**Published:** 2025-05-13

**Authors:** Jing Zhang, Qin-Bing Zhu, Yu-Sha Zeng, Ya-Hong Xue

**Affiliations:** ^1^Graduate School, Nanjing University of Chinese Medicine, Nanjing, China; ^2^Anorectal, Nanjing Hospital of Chinese Medicine Affiliated to Nanjing University of Chinese Medicine, Nanjing, China

**Keywords:** biofeedback, low anterior resection syndrome, bowel function, meta-analysis, LARS

## Abstract

**Background:**

As a series of bothersome bowel dysfunction symptoms, low anterior resection syndrome (LARS) has a high incidence after rectal cancer surgery, which grievously impairs health-related quality of life. There have been an increasing number of studies on biofeedback therapy (BFT) to recover intestinal function in patients following anus-preserving surgery. However, few systematic reviews or meta-analyses have been reported.

**Objective:**

The purpose of this systematic review with meta-analysis was to identify the short-term and long-term effects of BFT on subjective and objective indicators of LARS.

**Methods:**

Randomized controlled trials (RCT) published in PubMed, Cochrane Library, Web of Science, Embase, Chinese National Knowledge Infrastructure (CNKI), Wan Fang Data, China Biology Medicine disc (CBM), and Wei Pu (VIP) database from January 2012 to June 2024 were systematically searched. In accordance with PRISMA guidelines, the pooled findings were examined by Review Manager version 5.4.

**Results:**

The review finally included 14 RCT studies, with a total of 1,126 relevant patients. The meta-analysis results showed that following BFT, the mean resting pressure of the anal canal (MD = 5.53; 95% CI: 2.57, 8.49; Z = 3.66; *p* = 0.0003), the mean initial rectal sensation threshold (MD = 3.04; 95% CI: 1.84, 4.24; Z = 4.96; *p* < 0.00001), and the incidence rate of LARS (RR = 0.42; 95% CI: 0.31, 0.57; Z = 5.60; *p* < 0.00001) in the BFT intervention group were significantly better than those in the control group, However, there was no statistically significant differences in the Memorial Sloan-Kettering Cancer Center (MSKCC) intestinal function questionnaire score (MD = 0.79; 95% CI: −0.35, 1.93; Z = 1.37; *p* = 0.17), the CCIS (Wexner incontinence score) (MD = −0.67; 95% CI: −2.12, 0.78; Z = 0.91; *p* = 0.36), the LARS score (MD = −2.35; 95% CI: −6.07, 1.37; Z = 1.24; *p* = 0.22) and Xu ZF et al. “Five points Ten scores” excellent rate (RR = 4.59; 95% CI: 0.37, 56.35; Z = 1.19; *p* = 0.23) between the two groups.

**Conclusion:**

Our systematic review and meta-analysis indicate that BFT may improve the mean resting pressure of the anal canal and the mean initial rectal sensation threshold, reducing the incidence rate of LARS. Still, high-quality studies are necessary to explore the BFT standard for LARS.

**Systematic review registration:**

PROSPERO: CRD42024519785, https://www.crd.york.ac.uk/PROSPERO/view/CRD42024519785.

## Introduction

Globally, colorectal cancer (CRC) has the third-highest prevalence and the second-highest mortality among malignant tumors (1). Benefiting from the continuous advancement of neoadjuvant chemoradiotherapy and surgical techniques (such as low anterior resection with total mesorectal excision), the 5 year survival rate of patients with CRC has improved significantly. Nevertheless, patients undergoing anus-preserving surgery experience a cluster of intestinal dysfunction symptoms which seriously affect the patients’ health-related quality of life (HRQOL). These symptoms are referred to as the low anterior resection syndrome (LARS). The primary manifestations of LARS are stool frequency, urgency, fecal incontinence, difficulty evacuating the bowl, and the accompanying adverse consequences of HRQOL (2). According to relevant studies, up to 80% of patients following anus-preserving surgery are negatively affected by this condition, but there is no standard treatment strategy (3).

During the past few years, with the stupendous evolution in the diagnosis and treatment of rectal cancer, the HRQOL in patients following anus-preserving surgery has become worthy of our attention. Furthermore, much more attention has been drawn to the treatment strategy of LARS. As an emerging cognitive behavior therapeutic, biofeedback therapy commonly uses biofeedback therapeutic apparatus to monitor and amplify various physiological processes of the human body. Through reception of feedback signals such as vision, hearing or touch, individuals can consciously adjust their involuntary physiological activities to achieve the purpose of prevention and treatment (4). The aim of pelvic floor biofeedback training is to enhance the strength, stability, and coordination of the pelvic floor muscles, and improve the sensory function of the rectum. At present, biofeedback therapy, as a mature rehabilitation tool, has become one of the best prevention and treatment options for LARS (5). Although domestic and foreign scholars have actively carried out randomized controlled trails (RCTs) to observe the effect of biofeedback therapy on LARS, most studies were single-center trials with small sample sizes, lacking sufficient representativeness. Therefore, we conducted a systematic review with meta-analysis of various outcomes after anus-preserving surgery, to clarify the effectiveness of biofeedback therapy on LARS and provide evidence-based medicine reference for further clinical exploration of effective intervention measures for patients with LARS.

## Methods

This systematic review with meta-analysis was conducted in accordance with the Preferred Reporting Items for Systematic Reviews and Meta-Analyses (PRISMA) guidelines (6), and the review protocol has been registered in advance (PROSPERO CRD42024519785).

### Inclusion criteria

(1) Study design (S): RCTs of the effectiveness of biofeedback therapy on LARS after anus-preserving surgery for rectal cancer.
(2) Participants (P): Patients who underwent anus-preserving surgery for rectal cancer.
(3) Intervention (I): The intervention method included biofeedback therapy. If an additional therapy was used in combination with biofeedback therapy, there was a maximum of one additional therapy, and the control group (C) included this additional therapy.
(4) Outcomes (O): 1. Mean resting pressure of the anal canal; 2. Mean initial rectal sensation threshold; 3. MSKCC intestinal function questionnaire score; 4. CCIS (Wexner incontinence score); 5. LARS score; 6. The incidence rate of LARS; 7. The quality of life; 8. Xu ZF et al. “Five points Ten scores” excellent rate (Xu ZF et al. “Five points Ten scores” includes five items, such as awareness of defecation (0 ~ 2 points), bowel control ability (0 ~ 2 points), sensory function (0 ~ 2 points), defecation frequency (0 ~ 2 points), and defecation period (0 ~ 2 points). And the sum of the scores is the score of defecation function. If the score ≥ 7, it is considered to be excellent in defecation function).

### Exclusion criteria

(1) Non-RCT studies such as retrospective case–control studies, cohort studies, case reports, and systematic reviews.
(2) Studies in which the intervention included biofeedback therapy but in combination with more than one additional treatment.
(3) Repeated publication of a similar project, incomplete information, low quality research and the studies for which we were unable to obtain the original text or failed to extract effective information.
(4) Outcome indicators did not include subjective assessment of defecation function.
(5) Unavailable long-term follow-up information. For preventive studies, the study duration less than 6 months, and for therapeutic studies without LARS score, the study duration less than 3 months.

### Search strategy

We systematically searched RCTs published in PubMed, Cochrane Library, Web of Science, Embase, Chinese National Knowledge Infrastructure (CNKI), Wan Fang Data, China Biology Medicine disc (CBM), and Wei Pu (VIP) database. In addition, we tracked domestic and foreign unpublished literature, conference papers, clinical trials and other grey literature to reduce the possibility of omission.

The publication date was limited to the period from January 2012 to June 2024. The search terms were combined with MeSH terms and Entry terms according to the requirements of different databases: low anterior resection syndrome, anterior resection syndrome, postoperative rectal cancer, resection for rectal cancer, Lars, Ars and biofeedback.

In the process of literature retrieval in the databases, we referred to the retrieved article information, constantly supplemented the synonymy search terms, and finally determined the search strategy after several searches and adjustments. To prevent the reduction of search sensitivity and the risk of bias, we did not limit the type of study (exclude in Embase). At the same time, we hoped to find all relevant literature on the effectiveness of biofeedback therapy on LARS by paying attention to the references of relevant review articles (Appendix 1).

### Study selection and date extraction

The initially retrieved articles were imported into NoteExpress software. After the duplicate studies were removed, two reviewers read the title, keywords and abstract of the remaining studies, respectively. If an article roughly met the inclusion criteria or might have an impact on the research analysis, its full text was downloaded. Then, the general information, intervention measures (e.g., whether combined with pelvic floor muscle training or not, intervention time, control group), main outcome indicators, conclusions and other significant contents were extracted. If several articles were published on different aspects of the same trial, the article with the most comprehensive data was included in our review after exhaustive comparison. The data were independently screened, extracted and cross-checked in accordance with the pre-designed inclusion criteria. Disagreement was resolved by consensus with the assistance of other reviewers.

### Quality assessment

The quality of the included RCTs was evaluated independently by two reviewers using the Cochrane risk-of-bias 2 (RoB2) tool. The following main aspects were evaluated: (1) randomization process; (2) deviations from intended interventions; (3) missing outcome data; (4) measurement of the outcomes; (5) selection of the reported results; (6) overall. Each indicator was assessed as “low risk of bias” or “some concerns” or “high risk of bias.” In accordance with the above criteria, we assessed the likelihood of bias in each study and classified the study as high-quality (grade A), medium-quality (grade B), or low-quality (grade C).

### Data analysis

The review was performed using Review Manager version 5.4 for statistical analysis. The relative risk (RR) was used as an effect indicator for dichotomous variables, while the mean difference (MD) was used as an effect indicator for continuous variables. The 95% confidence intervals (CIs) were calculated, and *p* values lower than 0.05 were considered statistically significant in all cases. The value of I^2^ was used to quantify the heterogeneity among studies. In case with I^2^ < 50%, the heterogeneity was considered meaningless and a fixed-effect model was used to combine effect quantities. By contrary, there was significant heterogeneity, and a random-effect model was selected to calculate the results. When significant heterogeneity was observed, we further analyzed the source of heterogeneity by subgroup analysis according to the follow-up time after anus-preserving surgery. Then, the funnel plot formed by the software was used to identify potential publication bias. Finally, a sensitivity analysis of the included studies was conducted to evaluate the stability of the results.

## Results

### Study selection and characteristics

**Figure 1:**
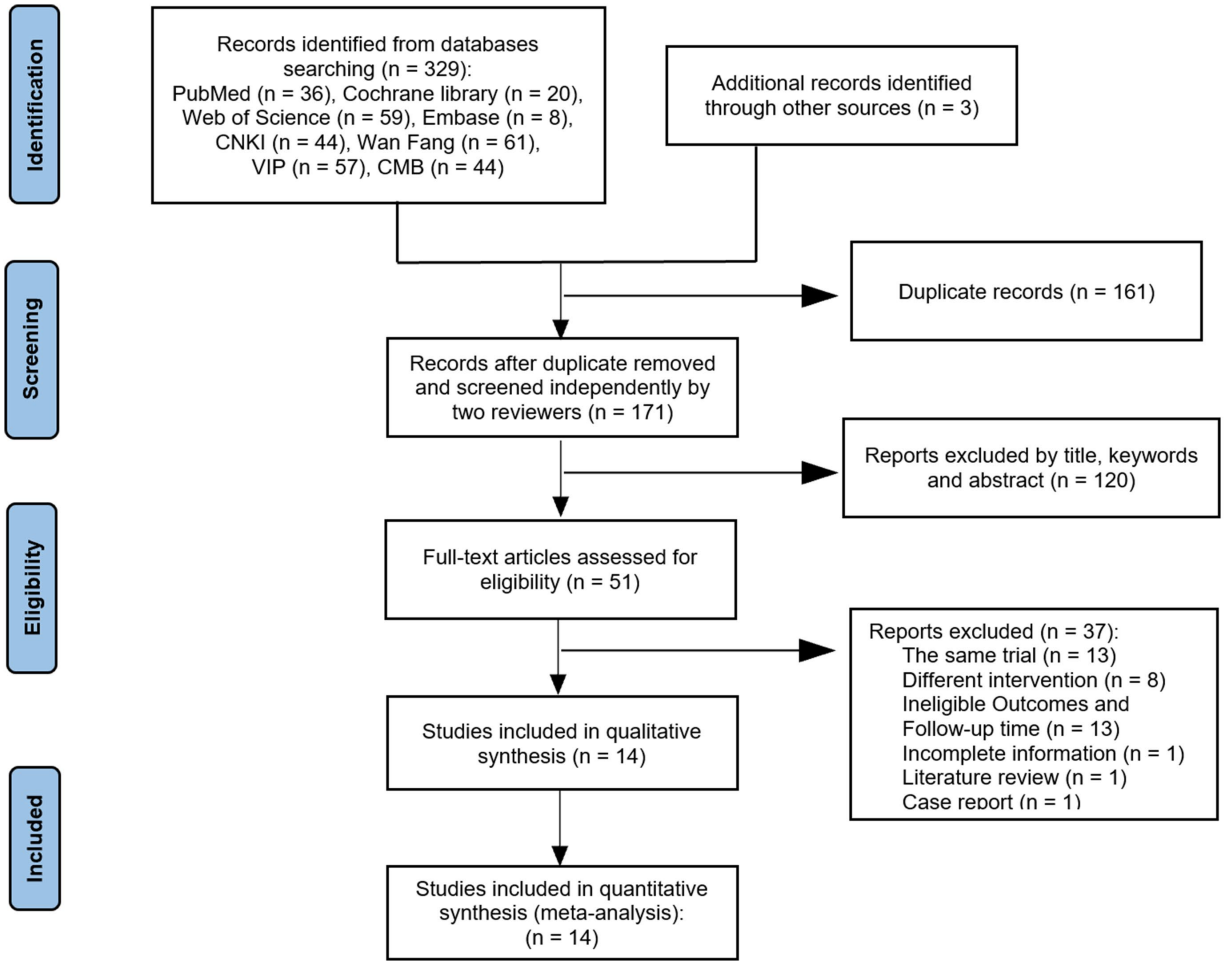
PRISMA flow diagram of the study selection process.

A total of 332 relevant studies were obtained through preliminary study selection (Figure 1). Based on the inclusion and exclusion criteria, two reviewers finally screened 14 (7–20) articles using NoteExpress software, including 12 Chinese articles (7–18) and 2 English articles (19, 20).

### Study characteristics and risk-of-bias evaluation

The subjects included in the 14 RCT articles were from China (7–18) and South Korea (19, 20), with a total of 1,126 relevant patients. The baselines of all studies were comparable, and the detailed basic characteristics were extracted (Tables 1, 2). The results of the quality assessment showed that there were only one article of grade A (7) and one article of grade C (19), whereas 12 articles were of grade B (7–11, 13–18, 20), belonging to the medium-quality studies (Table 3).

**Table 1:** Participants’ characteristics in the included studies.

Author and year	Sex (M/F)	Age (years)	Surgery type	Tumor height (cm)	Anostomosis height (cm)	Adjuvant therapy	Ostomy
Lee et al. (2019) Korea (19) *N* = 31	BFT group M:7 (43.8%) F:9 (56.2%) Control group M:12 (80.0%) F:3 (20.0%)	BFT group <70:14 (87.5%) ≥70:2 (12.5%) Control group <70:9 (60.0%) ≥70:6 (40.0%)	BFT group LAR:13 (81.2%) ISR:3 (18.8%) Control group LAR:14 (93.3%) ISR:1 (6.7%)	BFT group <5:8 (50.0%) ≥5:8 (50.0%) Control group <5:1 (6.7%) ≥5:14 (93.3%)	BFT group <5:13 (81.2%) ≥5:3 (18.8%) Control group <5:5 (33.3%) ≥5:10 (66.7%)	BFT group Yes:5 (31.2%) No:11 (68.8%) Control group Yes:1 (6.7%) No:14 (93.3%)	BFT group Yes:10 (62.5%) No:6 (37.5%) Control group Yes:2 (13.3%) No:13 (86.7%)
Kye et al. (2016) Korea (20) *N* = 47	BFT Group M:10 (47.6%) F:11 (52.4%) Control Group M:15 (57.7%) F:11 (42.3%)	BFT Group ≤65:13 (61.9%) >65:8 (38.1%) Control Group ≤65:13 (50.0%) >65:13 (50.0%)	**SPS**	BFT Group ≤5:6 (28.6%) >5:15 (71.4%) Control Group ≤5:8 (30.8%) >5:18 (69.2%)	BFT Group <5:6 (28.6%) ≥5:15 (71.4%) Control Group <5:3 (13.0%) ≥5:20 (87.0%) (according to the Period 5)	**nCRT**	**ALL**
Shi et al. (2023) China (11) *N* = 60	BFT Group M:21 (70.0%) F:9 (30.0%) Control Group M:19 (63.3%) F:11 (36.7%)	BFT Group 66.38 ± 4.93 Control Group 66.72 ± 4.86	**LAR**	**<6**	**NR**	**NR**	**NR**
Zhu et al. (2022) China (13) *N* = 110	BFT Group M:35 (63.6%) F:20 (36.4%) Control Group M:36 (65.5%) F:19 (34.5%)	BFT Group 59.72 ± 9.33 Control Group 60.26 ± 10.04	**LAR**	**NR**	**NR**	**nCRT**	**ALL**
Li et al. (2022) China (18) *N* = 86	BFT Group M:23 (53.5%) F:20 (46.5%) Control Group M:25 (58.1%) F:18 (41.9%)	BFT Group 52.49 ± 5.06 Control Group 52.52 ± 4.74	**NR**	**<8**	**NR**	**NR**	**NR**
Zhang (2021) China (25) *N* = 80	BFT Group M:18 (45.0%) F:22 (55.0%) Control Group M:24 (60.0%) F:16 (40.0%)	BFT Group 52.84 ± 8.15 Control Group 53.37 ± 8.56	**LAR**	**NR**	**NR**	**NR**	**NR**
Zhang (2021) China (14) *N* = 100	BFT Group M:24 (48.0%) F:26 (52.0%) Control Group M:25 (50.0%) F:25 (50.0%)	BFT Group 53.14 ± 8.39 Control Group 54.43 ± 9.78	**NR**	**NR**	**NR**	**NR**	**ALL**
Xu et al. (2021) China (12) *N* = 76	BFT Group M:27 (71.1%) F:11 (28.9%) Control Group M:26 (68.4%) F:12 (31.6%)	BFT Group 55.29 ± 4.40 Control Group 55.42 ± 4.12	**NR**	**NR**	**NR**	**NR**	**NR**
Peng et al. (2021) China (10) *N* = 82	BFT Group M:27 (65.9%) F:14 (34.1%) Control Group M:28 (68.3%) F:13 (31.7%)	BFT Group 53.22 ± 10.15 Control Group 52.88 ± 10.34	**Dixon**	BFT Group 5.04 ± 1.45 Control Group 5.06 ± 1.42	**NR**	**NR**	**ALL**
Wu et al. (2021) China (9) *N* = 120	BFT Group M:31 (51.7%) F:29 (48.3%) Control Group M:32 (53.3%) F:28 (46.7%)	BFT Group 55.21 ± 5.89 Control Group 55.13 ± 5.94	**NR**	BFT Group 4.10 ± 0.35 Control Group 4.05 ± 0.32	**NR**	**NR**	**NR**
Xu et al. (2020) China (16) *N* = 87	BFT Group M:31 (68.9%) F:14 (31.1%) Control Group M:25 (59.5%) F:17 (40.5%)	BFT Group 57.16 ± 8.21 Control Group 56.98 ± 8.00	**LAR**	BFT Group 4.21 ± 0.49 Control Group 4.16 ± 0.92	**NR**	**NO**	**NR**
Yang et al. (2020) China (15) *N* = 64	BFT Group M:21 (65.6%) F:11 (34.4%) Control Group M:21 (65.6%) F:11 (34.4%)	BFT Group 61.31 ± 10.05 Control Group 61.26 ± 10.05	**NR**	**NR**	**NR**	**NR**	BFT Group Yes:10 (31.3%) No:22 (68.7%) Control Group Yes:9 (28.1%) No:23 (71.9%)
Wu et al. (2019) China (7) *N* = 109	BFT Group M:25 (71.4%) F:10 (28.6%) PFMT Group M:27 (75.0%) F:9 (25.0%) Control Group M:30 (78.9%) F:8 (21.1%)	BFT Group 54.3 ± 9.9 PFMT Group 52.5 ± 10.4 Control Group 51.2 ± 12.3	**Dixon**	BFT Group 5.0 ± 1.6 PFMT Group 5.1 ± 1.9 Control Group 5.1 ± 1.9	**NR**	**nCRT**	**ALL**
Ni et al. (2018) China (8) *N* = 74	BFT Group M:30 (65.2%) F:16 (34.8%) Control Group M:19 (67.9%) F:9 (32.1%)	BFT Group 62.9 ± 8.6 Control Group 62.7 ± 6.7	**LAR**	BFT Group 4.0 ± 0.9 Control Group 3.9 ± 0.8	**NR**	**NO**	**NR**

M, male; F, female; BFT, biofeedback therapy; Dixon/LAR, low anterior resection; ISR, intersphincteric resection; SPS, sphincter-preserving surgery; nCRT, neoadjuvant chemoradiation therapy; PFMT, pelvic floor muscle training; NR, not reported.

**Table 2:** Characteristics of intervention, control, and outcomes of the included studies.

Author and year	Simple size (I/C)	Intervention		Treatment timing	BFT duration	Outcomes	Adverse events	Conclusion
		I group	C group					
Lee et al. (2019) Korea (19) *N* = 31	16/15	BFT and Loperamide	Observation with Loperamide	At least 2 months after surgery	Twice per week for a total of 10 times	①④⑤	NR	Although functional recovery of internal sphincter is hard to be induced by short-term rehabilitation, biofeedback therapy was superior for objective improvement of plevic function and LARS score to observation in LARS.
Kye et al. (2016) Korea (20) *N* = 47	21/26	BFT and Kegel (use Loperamide when necessary)	Kegel (use Loperamide when necessary)	During the temporary stoma interval	1 or 2 times a week	④	Anastomosis leak: 1 Postoperative anal strictures: 3	Although BFT have no effect on preventing anorectal dysfunction, it might be helpful for maintaining resting anal sphincter tone.
Shi et al. (2023) China (11) *N* = 60	30/30	BFT and Kegel	Kegel	1 week after surgery	20 min/once, 3/week, a total of 9 weeks	①②⑤	NR	BFT improves resting pressure of anal canal and initial rectal secsation threshold, alleviating the severity of LARS.
Zhu et al. (2022) China (13) *N* = 110	55/55	BFT and PFMT	PFMT	After inclusion in the study	20 min/once, once every 3 days, a total of 4 months divided into four stages	⑥	NR	BFT reduces the incidence rate of LARS.
Li et al. (2022) China (18) *N* = 86	43/43	BFT and PFMT	PFMT	Before the surgery	20 min/once, 2/week, a total of 6 months	①②⑥	NR	BFT improves resting pressure of anal canal and initial rectal secsation threshold, reducing the incidence rate of LARS.
Zhang (2021) China (25) *N* = 80	40/40	BFT and PFMT	PFMT	After LARS occurs	20 min/once, 3/week, a total of 4 months	④	NR	BFT improves postoperative intestinal function and alleviates the symptoms of LARS.
Zhang (2021) China (14) *N* = 100	50/50	BFT and PFMT	PFMT	Before the surgery	20 min/once, 3/week, a total of 4 months	⑥	ALL	BFT reduces the incidence rate of LARS.
Xu et al. (2021) China (12) *N* = 76	38/38	BFT and usual care	PFMT and usual care	After the surgery	20–25 min/once, 3/week	①⑥	NR	BFT improves resting pressure of anal canal and reduces the incidence rate of LARS.
Peng et al. (2021) China (10) *N* = 82	41/41	BFT and PFMT	PFMT	After the surgery	20 min/once, 3/week, a total of 4 months	③	NR	BFT improves postoperative intestinal function.
Wu et al. (2021) China (9) *N* = 120	60/60	BFT and PFMT	PFMT	1 month after surgery	Perianal muscle weakness: 20–30 min/once, 3/week, a total of 3 months Dysfunction of rectal sensation: 1 h/once, 2/week, a total of 3 months Incongruity of external sphincter muscle: 30 min/once, 3/week, a total of 3 months	①②③	NR	BFT improves postoperative resting pressure of anal canal, initial rectal secsation threshold and intestinal function.
Xu et al. (2020) China (16) *N* = 87	45/42	BFT, PFMT and usual care	PFMT and usual care	1 month after surgery	Perianal muscle weakness: 20 min/once, 3/week, a total of 3 months Dysfunction of rectal sensation: 1 h/once, 2/week, a total of 3 months Incongruity of external sphincter muscle: 30 min/once, 3/week, a total of 3 months	①②⑧	Anastomosis leak: 4	BFT improves postoperative resting pressure of anal canal, initial rectal secsation threshold and intestinal function.
Yang et al. (2020) China (15) *N* = 64	32/32	BFT and PFMT	PFMT	Before the surgery	20 min/once 3/week, a total of 4 months	①⑥⑦	NR	BFT improves postoperative resting pressure of anal canal, initial rectal secsation threshold, intestinal function and quality of life, reducing the incidence rate of LARS.
Wu et al. (2019) China (7) *N* = 109	35/36/38	BFT, PFMT and usual care	PFMT and usual care/usual care	After inclusion in the study	20 min/once, 3/week, a total of 4 months divided into four stages	①②③⑥	Anastomosis leak: 2	BFT improves postoperative resting pressure of anal canal, initial rectal secsation threshold and intestinal function, reducing the incidence rate of LARS.
Ni et al. (2018) China (8) *N* = 74	46/28	BFT	Observation	2 weeks after surgery	Strengthen perianal muscle weakness: 30 min/once, 1–2/day, a total of 3 months High rectal sensation threshold: NR Incongruity of external sphincter muscle and rectal sensation: 30 min/once, 1/day, a total of 3 months BFT with electrical stomulation: 15 min/once, 1/day, a total of 3 months	①②⑧	Anastomosis leak: 3 Local recurrence: 1	BFT improves postoperative resting pressure of anal canal, initial rectal secsation threshold and intestinal function.

I, intervention group; C, control group; BFT, biofeedback therapy; PFMT, pelvic floor muscle training; NR, not reported; LARS, low anterior resection syndrome. Outcomes: ① Mean resting pressure of the anal canal; ② Mean initial rectal sensation threshold; ③ MSKCC intestinal function questionnaire score; ④ CCIS (Wexner incontinence score); ⑤ LARS score; ⑥ Incidence rate of LARS; ⑦ Quality of life; ⑧Xu ZF et al. “Five points Ten scores” excellent rate.

**Table 3:** Risk of bias summary.

Ref	D1	D2	D3	D4	D5	Overall
Wu XD, 2019 (7)	**+**	**+**	**+**	**+**	**+**	**+**
Kye BH, 2016 (20)	**+**	**+**	**?**	**+**	**+**	**!**
Lee KH, 2019 (19)	**−**	**?**	**?**	**+**	**+**	**−**
Zhang H, 2021 (25)	**?**	**+**	**+**	**+**	**+**	**+**
Xu ZZ, 2021 (12)	**?**	**+**	**+**	**+**	**+**	**!**
Wu QH, 2021 (9)	**?**	**+**	**+**	**+**	**+**	**!**
Zhu XJ, 2022 (13)	**?**	**+**	**+**	**+**	**+**	**+**
Shi Y, 2023 (11)	**?**	**+**	**+**	**+**	**+**	**+**
Li J, 2022 (18)	**?**	**+**	**+**	**+**	**+**	**+**
Xu YF, 2020 (16)	**?**	**+**	**?**	**+**	**+**	**!**
Zhang H, 2021 (14)	**−**	**+**	**+**	**+**	**+**	**!**
Peng ZY, 2021 (10)	**?**	**+**	**+**	**+**	**+**	**+**
Yang JM, 2020 (15)	**?**	**+**	**+**	**+**	**+**	**+**
Ni XF, 2018 (8)	**?**	**+**	**+**	**+**	**+**	**+**

+: Low risk of bias; !&?: Some concerns; −: High risk-of-bias. D1: Randomization process; D2: Deviation from the intended interventions; D3: Missing outcome data; D4: Measurement of the outcome; D5: Selection of the reported results.

### Mean resting pressure of the anal canal

**Figure 2:**
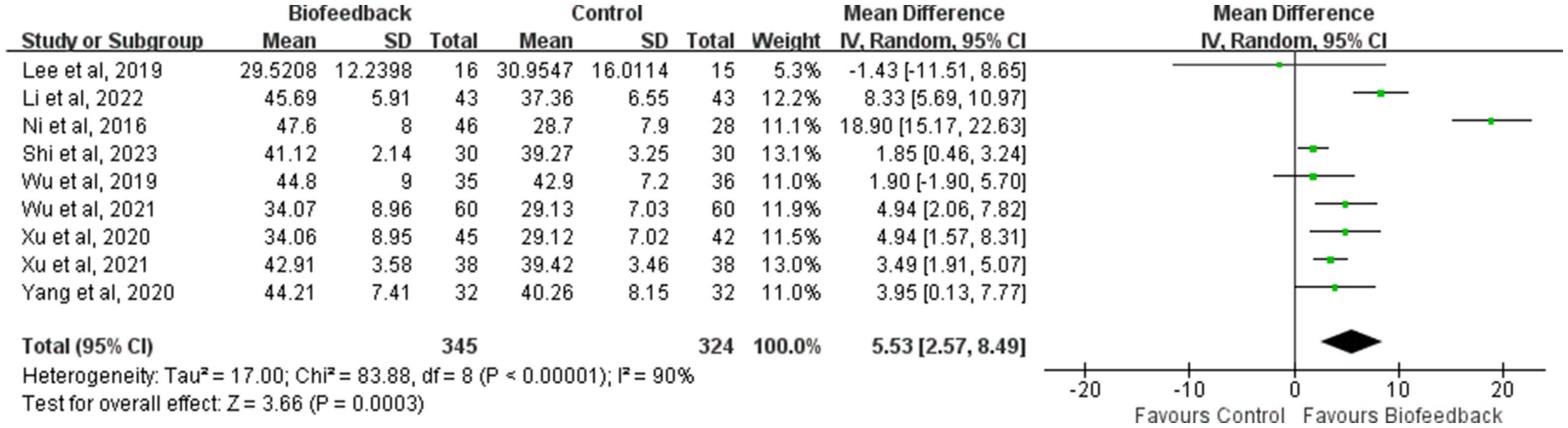
Meta-analysis of the mean resting pressure of the anal canal.

Nine studies (8, 9, 11–14, 17–19) reported the mean resting pressure of the anal canal of 345 patients with anus-preserving surgery after biofeedback therapy. The heterogeneity test results (*p* < 0.00001, I^2^ = 90%) suggested that there were differences among various studies, and a random-effect model was used for meta-analysis. The results of the meta-analysis showed that the mean resting pressure of the anal canal in the BFT group was significantly higher than that in the control group (MD = 5.53; 95% CI: 2.57, 8.49; Z = 3.66; *p* = 0.0003, Figure 2). Ni et al. (8) suggested that the significant difference in the mean resting pressure between the two groups could be related to the intervention time (a total of 3 months), follow-up time (12 months after intervention), and the control group with observational measure. By contrast, Lee et al. (19) considered that short-term rehabilitation was difficult to induce functional recovery of the internal sphincter (a total of 10 times).

### Mean initial rectal sensation threshold

**Figure 3:**
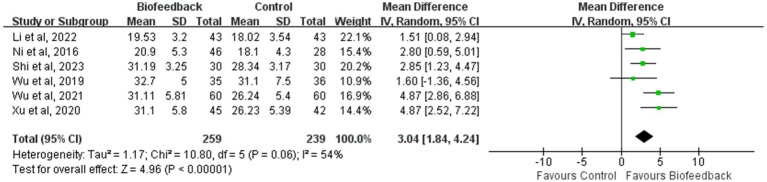
Meta-analysis of the initial rectal sensation threshold.

Six studies (8, 9, 11–14) reported the mean initial rectal sensation threshold of 498 patients with anus-preserving surgery after biofeedback therapy. The results of the meta-analysis showed that the mean initial rectal sensation threshold in the BFT group was significantly higher than that in the control group (MD = 3.04; 95% CI: 1.84, 4.24; Z = 4.96; *p* < 0.00001, Figure 3). However, considerable heterogeneity was detected (*p* = 0.06, I2 = 54%).

### MSKCC intestinal function questionnaire score

**Figure 4:**
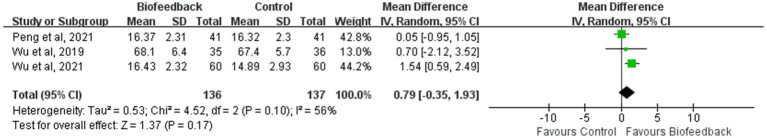
Meta-analysis of the MSKCC intestinal function questionnaire score.

Three studies (7, 9, 10) reported the MSKCC intestinal function questionnaire score of 273 patients with anus-preserving surgery after biofeedback therapy. The heterogeneity test results (*p* = 0.10, I^2^ = 56%) suggested that there were differences among various studies, and a random-effect model was used for meta-analysis. The results of the meta-analysis showed that the MSKCC intestinal function questionnaire score between the BFT group and the control group were comparable (MD = 0.79; 95% CI: −0.35, 1.93; Z = 1.37; *p* = 0.17, Figure 4).

### CCIS (Wexner incontinence score)

**Figure 5:**
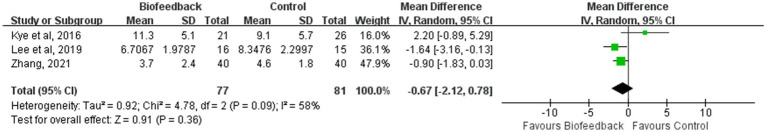
Meta-analysis of the CCIS (Wexner incontinence score).

Three studies (7, 19, 20) reported the CCIS of 158 patients with anus-preserving surgery after biofeedback therapy. With substantial heterogeneity between studies (*p* = 0.09, I^2^ = 58%), a random-effect model was used for meta-analysis. The results of the meta-analysis showed that there was no statistically significant difference in the CCIS between the BFT group and the control group (MD = −0.67; 95% CI: −2.12, 0.78; Z = 0.91; *p* = 0.36, Figure 5). Kye et al. (20) suggested that BFT during the temporary stoma interval had no effect on preventing fecal incontinence after temporary stoma reversal at 6 months.

### LARS score

**Figure 6:**
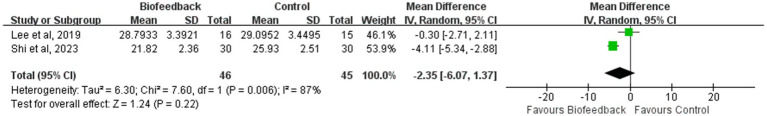
Meta-analysis of the LARS score.

Two studies (11, 19) reported the LARS score of 101 patients with anus−preserving surgery after biofeedback therapy. The heterogeneity test results (*p* = 0.006, I^2^ = 87%) suggested that there were differences among various studies, and a random-effect model was used for meta-analysis. The results of the meta-analysis showed that there was no statistically significant difference in the LARS score between the BFT group and the control group (MD = −2.35; 95% CI: −6.07, 1.37; Z = 1.24; *p* = 0.22, Figure 6). It is undeniable that most of the patients transition from Major LARS to Minor LARS.

### The incidence rate of LARS

**Figure 7:**
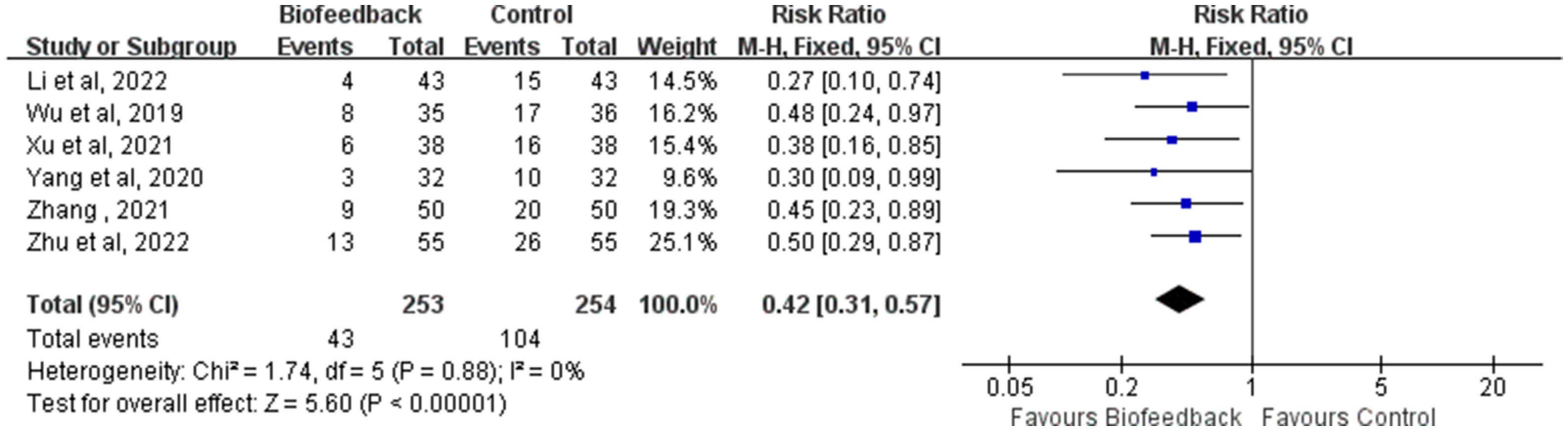
Meta-analysis of the incidence rate of LARS.

Six studies (12–15) reported the incidence rate of LARS in 507 patients with anus-preserving surgery after biofeedback therapy. The heterogeneity test results (*p* = 0.88, I^2^ = 0%) suggested that there were no differences among various studies, and a fixed-effect model was used for meta-analysis. BFT significantly decreased the incidence rate of LARS compared to control (RR = 0.42; 95% CI: 0.31, 0.57; Z = 5.60; *p* < 0.00001, Figure 7). The incidence rate of LARS in the BFT group was 0.41 times that of the control group.

### Xu ZF et al. “five points ten scores” excellent rate

**Figure 8:**
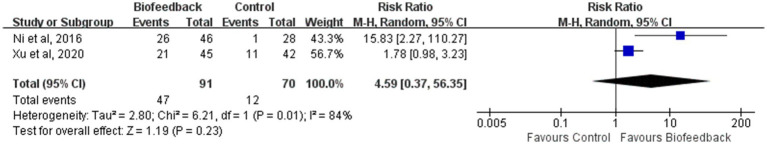
Meta-analysis of the Xu ZF et al. “Five points Ten scores” excellent rate.

Two studies (8, 16) reported Xu ZF et al. “Five points Ten scores” excellent rate of 161 patients with anus-preserving surgery after biofeedback therapy. With substantial heterogeneity between studies (*p* = 0.01, I^2^ = 84%), a random-effect model was used for meta-analysis. There was no statistically significant difference in Xu ZF et al. “Five points Ten scores” excellent rate between the BFT group and the control group (RR = 4.59; 95% CI: 0.37, 56.35; Z = 1.19; *p* = 0.23, Figure 8).

### The quality of life

Only one study (15) reported the quality of life of 64 patients with anus-preserving surgery after biofeedback therapy. The SF-36 (Short Form 36 Health Survey) was used. The results showed that the quality of life in the BFT group at 3 months after surgery was higher than that before the surgery, whereas the quality of life in the control group was lower than that before the surgery. Compared with the control group, BFT can significantly improve the overall quality of life.

### Subgroup analysis

**Figure 9:**
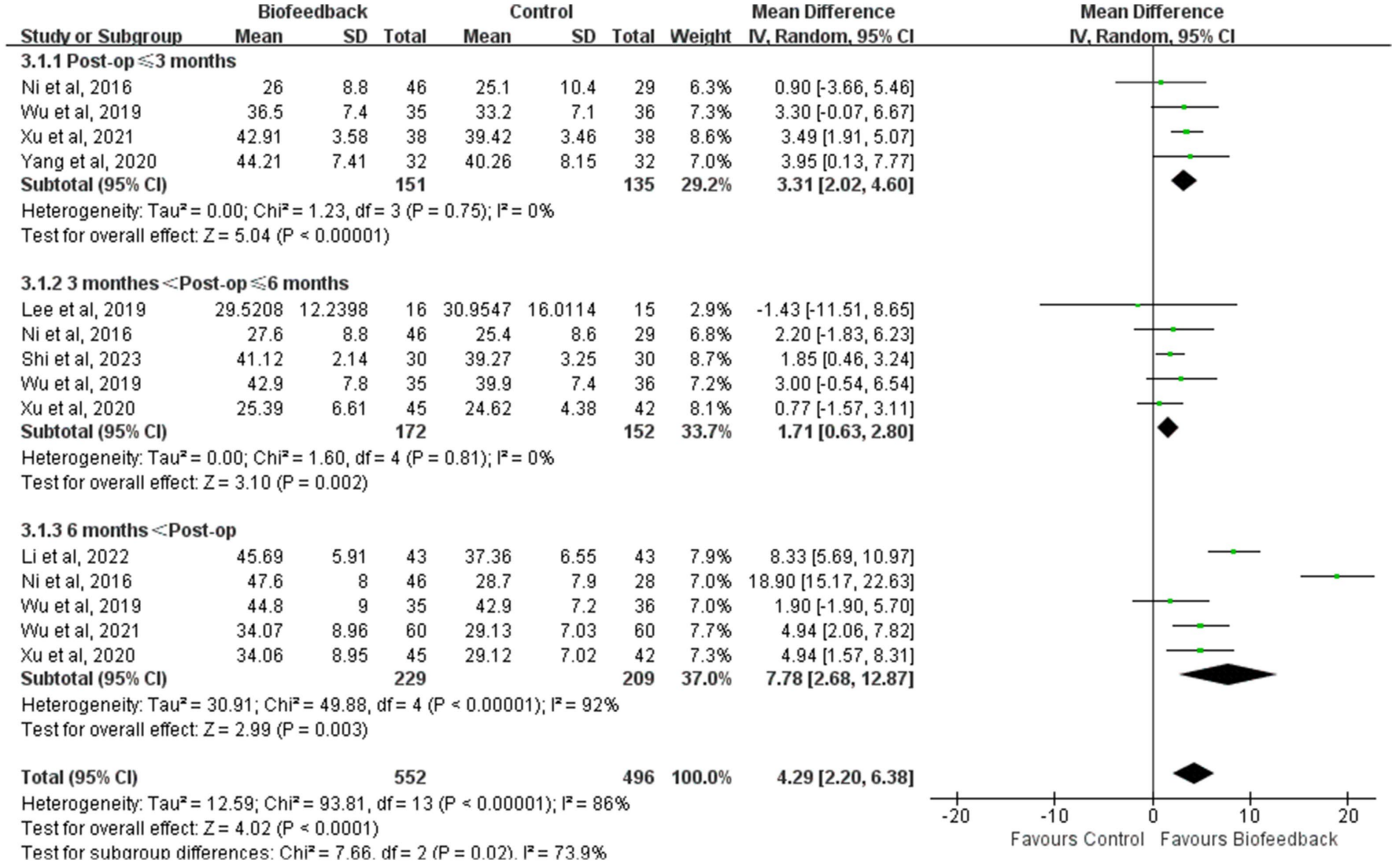
Subgroup-analysis of the mean resting pressure of the anal canal.

In view of the limited number of studies screened, the different outcomes in each study, and the lack of partial data, subgroup analysis was conducted to examine the mean resting pressure of the anal canal and the incidence rate of LARS according to the follow-up time after anus-preserving surgery. Subgroup analysis results revealed that compared with the control group, the BFT group showed a higher mean resting pressure of the anal canal (*p* < 0.00001; *p* = 0.002; *p* = 0.003, Figure 9) and a lower incidence rate of LARS (Supplementary Figure S1) in three different periods. Although the degree of difference on the certain period was not obvious, it was statistically significant. We found that the mean resting pressure of the anal canal decreased in the early postoperative period and then gradually recovered stability. At the follow-up period between 3 and 6 months, the differences in the mean resting pressure and the incidence rate of LARS between the two groups were minimal. This might be related to the fact that major LARS occurring 3–6 months after surgery results in persistent intestinal dysfunction (17). Changes in heterogeneity between the groups were reflected by I^2^. Considering that the control group from Ni et al. (8) and the intervention time from Li et al. (18) were significantly different from those in other studies, we removed two studies in the follow-up period of more than 6 months. This eliminated the difference between different periods. Hence, the follow-up time may be a source of heterogeneity.

### Publication bias and sensitivity analysis

In our review, publication bias was not assessed using the mean resting pressure of anal canal or the incidence rate of LARS due to the limited number of included studies.

Using the mean resting pressure of the anal canal and the incidence rate of LARS as primary outcome indexes, sensitivity analysis was conducted by sequentially excluding one of the included studies. We found that there was no influence on the incidence rate of LARS, and the analyses were robust. As for the mean resting pressure of the anal canal, the exclusion of the study by Ni et al. (8) had a significant impact on the heterogeneity test results (*p* = 0.004, I2 = 67%), but the results of the meta-analysis still showed that the mean resting pressure of the anal canal was significantly higher than that in the control group (Supplementary Figure S2).

## Discussion

The international consensus previously defined LARS as the disordered bowel function after rectal resection, leading to a detriment in quality of life (21). This broad definition is mainly based on patients’ subjective perception, which does not allow the precise measurement of LARS. Thus, the eight symptom complexes and eight consequences were developed to be of the highest priority when defining LARS (2). According to Chen et al. (22), the intestinal function of LARS patients was severely impaired. Namely, the objective indexes of resting anal pressure, squeezing pressure, urge volume and rectal maximum volume threshold were significantly lower than those in normal or fecal incontinence (FI) patients. Taken together, the 14 RCTs included in this review were evaluated from the following eight aspects to systematically explore the evidence for the effectiveness of biofeedback therapy on LARS: 1. mean resting pressure of the anal canal; 2. mean initial rectal sensation threshold; 3. MSKCC intestinal function questionnaire score; 4. CCIS; 5. LARS score; 6. the incidence rate of LARS; 7. the quality of life; 8. Xu ZF et al. “Five points Ten scores” excellent rate.

Considering that biofeedback therapy requires the cooperation of participants, the blinding is difficult to implement. Accordingly, although the blinding was unclear, 1 study (7) had a low risk of bias, 1 study (19) had a high risk of bias and 12 studies (7–11, 13–18, 20) had a moderate risk of bias. In the study by Lee et al. (19), patients who accepted biofeedback therapy were enrolled in the intervention group, which may lead patients with more severe symptoms to seek biofeedback therapy. While Zhang et al. (14) divided patients into two groups according to the order of their visit. These two studies had a high risk on the item of the randomization process. When comparing anorectal manometry (ARM) between the studies of Xu et al. (16) and Wu et al. (9), we found that the data in the two studies were extremely similar. Therefore, we believe that Wu et al. (9) had a high risk of the other bias. This reflects that the design of the RCTs included in the review was not rigorous.

Previous systematic reviews (23, 24) have reported the effectiveness of biofeedback therapy in patients with bowel dysfunction following rectal cancer surgery. Li and colleagues (24) reviewed 2 prospective randomized trials, 2 prospective non-randomized trials and 8 case series. They found that biofeedback therapy can significantly promote the recovery of physiological intestinal function, alleviate the clinical symptoms of fecal incontinence, and comprehensively improve the postoperative quality of life. However, these reviews lack RCTs. Consequently, our review incorporated more RCTs.

The biggest advantage of biofeedback is that it allows patients to intuitively feel the changes in anorectal dynamics, to continuously strengthen neural reflex pathway and regulate disordered muscle activity. Similarly, our review also confirmed that BFT could improve the mean resting pressure of the anal canal and the mean initial rectal sensation threshold, and reduce the incidence rate of LARS. Among the included studies, 9 studies (8, 9, 11–14, 17–19) reported the mean resting pressure of the anal canal, 6 studies (8, 9, 11–14) reported the mean initial rectal sensation threshold and 6 studies (12–15) reported the incidence rate of LARS. After combining effect quantities, the mean resting pressure of the anal canal and the mean initial rectal sensation threshold in the BFT group were higher than those in the control group, whereas the incidence of LARS was lower than that in the control group.

Nevertheless, there were no statistically significant differences in the results of the subjective assessment, such as MSKCC intestinal function questionnaire score, CCIS, LARS score, the quality of life and Xu ZF et al. “Five points Ten scores” excellent rate. Because of the lack of a recognized unified evaluation standard for LARS in clinical practice, each center has its own evaluation system. As a result, the number of included studies was relatively small when combining effect quantities of such outcomes. Therefore, future research should not only apply anorectal manometry to objectively assess intestinal function, but also pay more attention to subjective bowel function (MSKCC intestinal function questionnaire score, CCIS, LARS scores) and the quality of life.

It is worth mentioning that we restricted the follow-up time of the included studies. The follow-up time for two therapeutic studies with a LARS score (11, 19) was 5 weeks and 9 weeks, while the follow-up time for one therapeutic study without a LARS score (25) was 7 months. In addition, the follow-up time for 11 preventive studies (8–10, 12–18, 20) ranged from 7 to 16 months after surgery. According to Qin et al. (26), bowel dysfunction after rectal cancer surgery is time-dependent. The severity of LARS is the most significant in the early postoperative period, and can be gradually relieved. Although LARS tends to be stable for 1–2 years after surgery, some symptoms can last for decades. It is hard to tell whether changes in intestinal function are the result of natural recovery or the positive effect of BFT. According to dynamic and continuous follow-up evaluations in our review, we believe that the long-term effectiveness of BFT can be maintained and the advantages of BFT become more prominent with the passage of postoperative time.

The duration of intervention in most of the studies ranged from 3 to 4 months, and the minimum intervention period for BFT was 5 weeks. The timing of BFT intervention varied among the studies. Namely, some studies started the intervention before the surgery, whereas others began BFT after the surgery. Therefore, additional research is needed to identify the optimal biofeedback treatment method, which can better facilitate postoperative recovery and prevent LARS.

Recent studies have indicated that neoadjuvant chemoradiation therapy and anastomosis height are independent risk factors for major LARS (27). However, given that some of the included studies (7–11, 15–18) did not report the data on these aspects, we were unable to perform subgroup analysis for these heterogeneity indicators. The pathophysiology of LARS includes a combination of multiple factors, such as colonic dysmotility, neorectal reservoir dysfunction, and anal sphincter dysfunction. To the best of our knowledge, anal sphincter dysfunction and neorectal reservoir dysfunction are closely related to incontinence-dominant symptoms, while colonic dysmotility and neorectal reservoir dysfunction are tightly related to frequency-dominant symptoms. According to Liu et al. (28), patients with rectal cancer who have survived more than 5 years after sphincter-preserving surgery still have a high prevalence of LARS. Incontinence-dominant symptoms can be recovered at 1 year after surgery. Conversely, frequency-dominant symptoms are not associated with the postoperative time. Biofeedback therapy has been recommended for the short-term and long-term treatment of fecal incontinence (29). The included studies mainly focused on the situation of fecal incontinence and drew a conclusion, consistent with the result of the consensus guidelines conducted by Rao et al. (29). Although BFT can also alleviate the urgency of defecation to a certain extent by sensory training, the existing evidence does not yet support this viewpoint. It is necessary to conduct more high-quality research to focus on the frequency-dominant symptoms.

## Limitation

Potential limitations of this review lie in the following aspects: 1. Different centers have different standards for measuring the objective intestinal function of ARM, resulting in a risk of bias in data consistency. 2. Due to the limited number of included studies, we did not assess publication bias using the mean resting pressure of the anal canal or the incidence rate of LARS. 3. Most studies on BFT for LARS are still single-center studies with a small sample size. In the process of study design, few studies explain the possible risk of bias in detail, such as those related to randomization, assignment, and blinding method. Moreover, the setting of outcomes is not comprehensive, and the overall quality of the literature is not high.

## Conclusion

Our systematic review and meta-analysis indicate that BFT may improve the mean resting pressure of the anal canal and the mean initial rectal sensation threshold, reducing the incidence rate of LARS. However, given the limitations of the included literature, the short-term and long-term effects of biofeedback on the improvement of total LARS symptoms (particularly on frequency-dominant symptoms) warrant further analysis. Still, prospective RCTs should be conducted in the future with a more rigorous study design, strict inclusion criteria, and detailed evaluation of the anastomosis height, neoadjuvant therapy, preventive ostomy, and complications affecting LARS to reduce intergroup heterogeneity. Furthermore, we assert that studies should try to avoid bias caused by random allocation and the combination of multiple means by formulating more reasonable intervention control measures. At the same time, more high-quality RCT studies with large samples (Multi-center collaboration), long-term (intervention for 4 weeks, assessment at 1 month and 6 months after the intervention, and 1-2 years follow-up) and comprehensive preoperative or postoperative evaluation indicators (LARS score, Anorectal Manometry, EORTC QLQ-CR29, Symptom management diary) are necessary to continuously strengthen the meta-analysis of the effectiveness of biofeedback on LARS, so that the comprehensive treatment standard for LARS can be applied to clinical practice more accurately.

## Data Availability

The original contributions presented in the study are included in the article/Supplementary material, further inquiries can be directed to the corresponding author.

## References

[1] Sung H Ferlay J Siegel RL Laversanne M Soerjomataram I Jemal A . Global cancer statistics 2020: GLOBOCAN estimates of incidence and mortality worldwide for 36 cancers in 185 countries. CA Cancer J Clin. (2021) 71:209–49. doi: 10.3322/caac.21660, PMID: 33538338

[2] Keane C Fearnhead NS Bordeianou LG Christensen P Basany EE Laurberg S . International consensus definition of low anterior resection syndrome. Dis Colon Rectum. (2020) 63:274–84. doi: 10.1097/DCR.0000000000001583, PMID: 32032141 PMC7034376

[3] Dulskas A Smolskas E Kildusiene I Samalavicius NE. Treatment possibilities for low anterior resection syndrome: a review of the literature. Int J Color Dis. (2018) 33:251–60. doi: 10.1007/s00384-017-2954-x, PMID: 29313107

[4] Narayanan SP Bharucha AE. A practical guide to biofeedback therapy for pelvic floor disorders. Curr Gastroenterol Rep. (2019) 21:21. doi: 10.1007/s11894-019-0688-3, PMID: 31016468

[5] Hite M Curran T. Biofeedback for pelvic floor disorders. Clin Colon Rectal Surg. (2021) 34:56–61. doi: 10.1055/s-0040-1714287, PMID: 33536850 PMC7843943

[6] Moher D Liberati A Tetzlaff J Altman DG The PRISMA Group. Preferred reporting items for systematic reviews and meta–analyses: the PRISMA statement. PLoS Med. (2009) 6:e1000097. doi: 10.1371/journal.pmed.1000097, PMID: 19621072 PMC2707599

[7] Wu XD Fu CF Chen YL Kong LH Pan ZZ Zheng MC. Intervention effect of biofeedback combined with pelvic floor muscle exercise on low anterior resection syndrome in patients with low anus–preserving rectal cancer. Nat Med J China. (2019) 99:2337–43. doi: 10.3760/cma.j.issn.0376-2491.2019.30.004, PMID: 31434413

[8] Ni XF Chai R Chen S . Study on anorectal dynamics of biofeedback therapy for fecal incontinence in patients with low rectal cancer after restorative resection. Chin J Exp Surg. (2018) 35:226–9. doi: 10.3760/cma.j.issn.1001-9030.2018.02.010

[9] Wu QH. Effect of individualized biofeedback training combined with pelvic floor muscle exercise on postoperative bowel function in patients with rectal Cancer undergoing anus preservation surgery. Reflexol Rehabil Med. (2021) 2:94–96,126 doi: 10.3969/j.issn.2096-7950.2021.7.fshlfykfyx202107028

[10] Peng ZY Peng M An LF . Effects of biofeedback training on postoperative intestinal function in patients with rectal Cancer anal preservation. Chin Foreign Med Res. (2021) 19:191–4. doi: 10.14033/j.cnki.cfmr.2021.23.067

[11] Shi Y Song GL Jiang CQ. Effect of biofeedback combined with Kegel training on rectal function recovery in patients after low anterior rectal resection. Med Innov China. (2023) 20:172–6. doi: 10.3969/j.issn.1674-4985.2023.25.040

[12] Xu ZZ Zhao LL Gao Y . Effects of biofeedback training on prevention of anterior resection syndrome in rectal cancer patients underwent an anus–preserving surgery. Health Guide. (2021) 25:44. Available at: https://d.wanfangdata.com.cn/periodical/Ch9QZXJpb2RpY2FsQ0hJTmV3UzIwMjUwMTE2MTYzNjE0EhF5c2Jqem4teDIwMjEyNTA0MBoIcWp3cnBiOWU%3D

[13] Zhu XJ Jia F. The efficacy of personalized biofeedback training combined with pelvic–floor muscles exercise for preventing anterior resection syndrome following the radical surgery for low rectal Cancer. Chin J Coloproctol. (2022) 42:53–4. doi: 10.3969/j.issn.1000-1174.2022.08.019

[14] Zhang H. Effects of biofeedback training on prevention of anterior resection syndrome in rectal cancer patients underwent an anus–preserving surgery. Med Hyg. (2021) 8:0269–70. Available at: https://qikan.cqvip.com/Qikan/Article/Detail?id=1000003103546&from=Qikan_Search_Index

[15] Yang JM Wang SX Wang ZX. The effect of biofeedback training combined with pelvic floor muscle exercise on anal and rectal function in patients with middle and low rectal cancer. J Clin Nurs. (2020) 19:51–3. doi: 10.3969/j.issn.1671-8933.2020.05.018

[16] Xu YF. Intervention effect of personalized biofeedback therapy for anorectal dynamics and defecation dysfunction in patients with rectal cancer after restorative resection. Chin J Pract Nurs. (2020) 36:1612–7. doi: 10.3760/cma.j.cn211501-20191111-03299

[17] Varghese C Wells CI O’Grady G Christensen P Bissett IP Keane C . The longitudinal course of low–anterior resection syndrome: An individual patient Meta–analysis. Ann Surg. (2022) 276:46–54. doi: 10.1097/SLA.0000000000005423, PMID: 35185131

[18] Li J Lei Y. Effects analysis of biofeedback training on prevention of low anterior resection syndrome and urinary dysfunction after radical resection for low rectal cancer. Guizhou Med J. (2022) 46:1741–2. doi: 10.3969/j.issn.1000-744X.2022.11.036

[19] Lee KH Kim JS Kim JY. Efficacy of biofeedback therapy for objective improvement of pelvic function in low anterior resection syndrome. Ann Surg Treat Res. (2019) 97:194–201. doi: 10.4174/astr.2019.97.4.194, PMID: 31620393 PMC6779952

[20] Kye BH Kim HJ Kim G Yoo RN Cho HM. The effect of biofeedback therapy on anorectal function after the reversal of temporary stoma when administered during the temporary stoma period in rectal Cancer patients with sphincter–saving surgery: the interim report of a prospective randomized controlled trial. Medicine (Baltimore). (2016) 95:e3611. doi: 10.1097/MD.0000000000003611 27149496 PMC4863813

[21] Bryant CL Lunniss PJ Knowles CH Thaha MA Chan CL. Anterior resection syndrome. Lancet Oncol. (2012) 13:e403–8. doi: 10.1016/S1470-2045(12)70236-X, PMID: 22935240

[22] Chen SC Futaba K Leung WW Wong C Mak T Ng S . Functional anorectal studies in patients with low anterior resection syndrome. Neurogastroenterol Motil. (2022) 34:e14208. doi: 10.1111/nmo.14208, PMID: 34145694

[23] Wang XY Wang JM. The efficacy of biofeedback training on low anterior resection syndrome after anal preservation for rectal cancer: a Meta–analysis. Chinese evidence–based. Nursing. (2024) 10:2099–105.

[24] Li H Guo C Gao J Yao H. Effectiveness of biofeedback therapy in patients with bowel dysfunction following rectal Cancer surgery: a systemic review with Meta–analysis. Ther Clin Risk Manag. (2022) 18:71–93. doi: 10.2147/TCRM.S344375, PMID: 35140468 PMC8819167

[25] Zhang H. Observation of 80 cases of biofeedback therapy on treatment of low anterior resection syndrome. Da Jian Kang. (2021) 21:156–7. Available at: https://d.wanfangdata.com.cn/periodical/Ch9QZXJpb2RpY2FsQ0hJTmV3UzIwMjUwMTE2MTYzNjE0EhpRS0JKQkQyMDIxMjAyMTA4MTMwMDAxNzY0MxoIdWtnMzlhZWM%3D

[26] Qin QY Hang BJ Wang L. Low anterior resection syndrome: current awareness, prevention, and treatment. Chin J Color Dis (Electronic Edition). (2016) 5:198–203. doi: 10.3877/cma.j.issn.2095-3224.2016.03.001

[27] Sakr A Sauri F Alessa M Zakarnah E Alawfi H Torky R . Assessment and management of low anterior resection syndrome after sphincter preserving surgery for rectal cancer. Chin Med J (Engl). (2020) 133:1824–33. doi: 10.1097/CM9.0000000000000852, PMID: 32604174 PMC7469998

[28] Liu F Hou S Gao ZD . Cross–sectional study of low anterior resection syndrome in patients who have survived more than 5 years after sphincter–preserving surgery for rectal cancer. Chin J Gastrointes Surg. (2023) 26:283–9. doi: 10.3760/cma.j.cn441530-20220914-00384 36925129

[29] Rao SS Benninga MA Bharucha AE Chiarioni G Di Lorenzo C Whitehead WE. ANMS–ESNM position paper and consensus guidelines on biofeedback therapy for anorectal disorders. Neurogastroenterol Motil. (2015) 27:594–609. doi: 10.1111/nmo.12520, PMID: 25828100 PMC4409469

