# Supplementary material for: Effectiveness of biofeedback therapy on low anterior resection syndrome: a systemic review with meta-analysis
Source: Front Med (Lausanne). 2025 May 13;12:1538114. doi: 10.3389/fmed.2025.1538114 (PMC12106574; doi:10.3389/fmed.2025.1538114)
Supplement: Supplementary file 1 [file Data_Sheet_1.pdf]

Study or Subgroup	Biofeedback Events	Biofeedback Total	Control Events	Control Total	Weight	Risk Ratio M-H, Fixed, 95% CI
**3.2.1 Post-op≤6 months**						
Li et al, 2022	2	43	9	43	4.0%	0.22 [0.05, 0.97]
Wu et al, 2019	15	35	21	36	9.3%	0.73 [0.46, 1.18]
Xu et al, 2021	12	38	19	38	8.5%	0.63 [0.36, 1.11]
Yang et al, 2020	12	32	13	32	5.8%	0.92 [0.50, 1.70]
Zhang , 2021	20	50	26	50	11.6%	0.77 [0.50, 1.18]
Zhu et al, 2022	21	55	32	55	14.3%	0.66 [0.44, 0.98]
**Subtotal (95% CI)**		**253**		**254**	**53.6%**	**0.69 [0.55, 0.85]**
Total events	82		120			
Heterogeneity: Chi² = 3.63, df = 5 (P = 0.60); I² = 0%						
Test for overall effect: Z = 3.45 (P = 0.0006)						
**3.2.2 6 months<Post-op≤9 months**						
Li et al, 2022	4	43	15	43	6.7%	0.27 [0.10, 0.74]
Xu et al, 2021	6	38	16	38	7.2%	0.38 [0.16, 0.85]
Yang et al, 2020	3	32	10	32	4.5%	0.30 [0.09, 0.99]
Zhu et al, 2022	13	55	26	55	11.6%	0.50 [0.29, 0.87]
**Subtotal (95% CI)**		**168**		**168**	**30.0%**	**0.39 [0.26, 0.58]**
Total events	26		67			
Heterogeneity: Chi² = 1.52, df = 3 (P = 0.68); I² = 0%						
Test for overall effect: Z = 4.69 (P < 0.00001)						
**3.2.3 9 months<Post-op**						
Wu et al, 2019	8	35	17	36	7.5%	0.48 [0.24, 0.97]
Zhang , 2021	9	50	20	50	8.9%	0.45 [0.23, 0.89]
**Subtotal (95% CI)**		**85**		**86**	**16.5%**	**0.47 [0.29, 0.76]**
Total events	17		37			
Heterogeneity: Chi² = 0.02, df = 1 (P = 0.88); I² = 0%						
Test for overall effect: Z = 3.07 (P = 0.002)						
**Total (95% CI)**		**506**		**508**	**100.0%**	**0.56 [0.47, 0.67]**
Total events	125		224			
Heterogeneity: Chi² = 12.88, df = 11 (P = 0.30); I² = 15%						
Test for overall effect: Z = 6.43 (P < 0.00001)						
Test for subgroup differences: Chi² = 7.15, df = 2 (P = 0.03), I² = 72.0%						

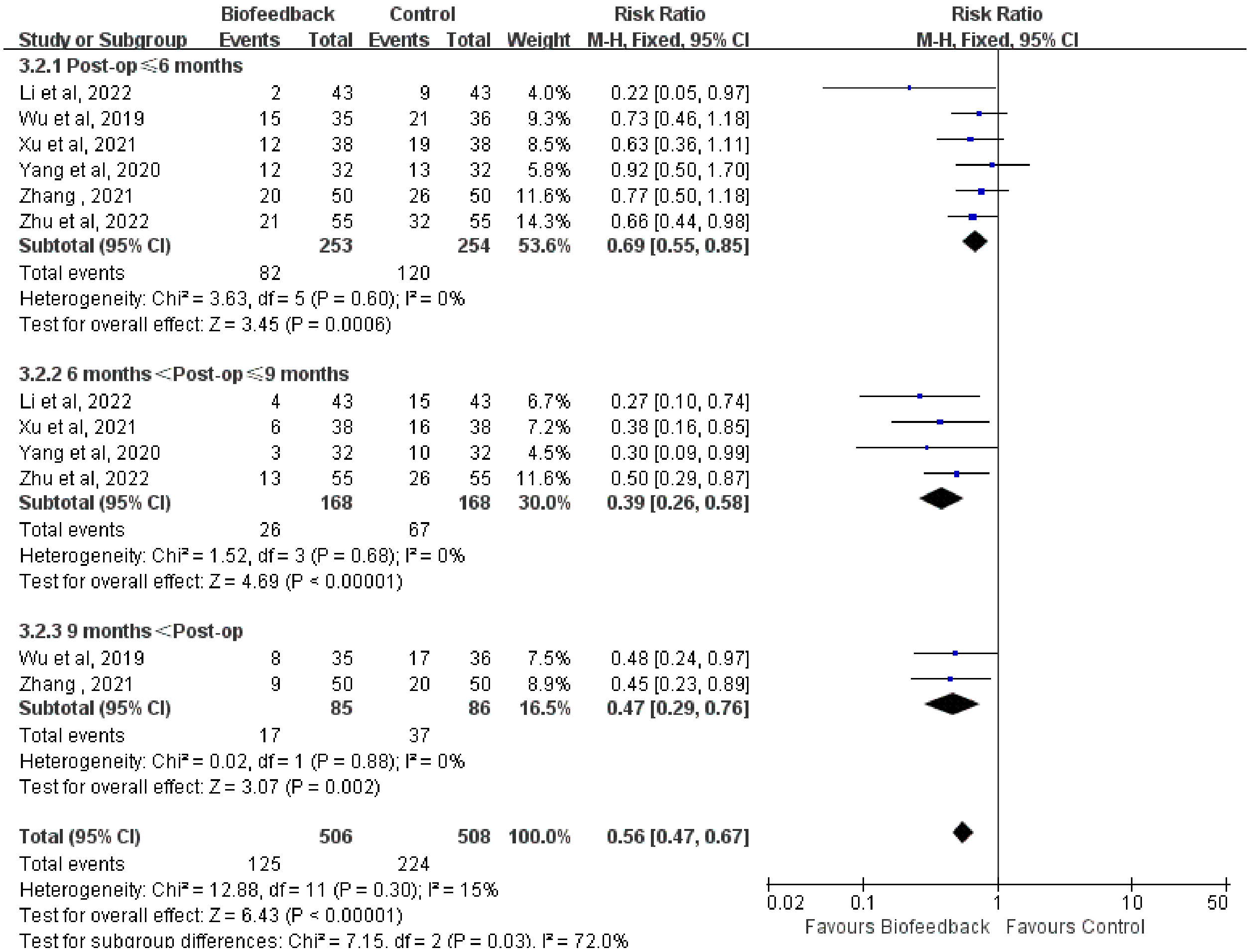

Figure S1 Subgroup−analysis of the incidence rate of LARS

Study or Subgroup	Biofeedback Mean	Biofeedback SD	Biofeedback Total	Control Mean	Control SD	Control Total	Weight	Mean Difference IV, Random, 95% CI
Lee et al, 2019	29.5208	12.2398	16	30.9547	16.0114	15	2.5%	-1.43 [-11.51, 8.65]
Li et al, 2022	45.69	5.91	43	37.36	6.55	43	14.3%	8.33 [5.69, 10.97]
Ni et al, 2016	47.6	8	46	28.7	7.9	28	0.0%	18.90 [15.17, 22.63]
Shi et al, 2023	41.12	2.14	30	39.27	3.25	30	18.9%	1.85 [0.46, 3.24]
Wu et al, 2019	44.8	9	35	42.9	7.2	36	10.5%	1.90 [-1.90, 5.70]
Wu et al, 2021	34.07	8.96	60	29.13	7.03	60	13.4%	4.94 [2.06, 7.82]
Xu et al, 2020	34.06	8.95	45	29.12	7.02	42	11.8%	4.94 [1.57, 8.31]
Xu et al, 2021	42.91	3.58	38	39.42	3.46	38	18.2%	3.49 [1.91, 5.07]
Yang et al, 2020	44.21	7.41	32	40.26	8.15	32	10.4%	3.95 [0.13, 7.77]
**Total (95% CI)**			**299**			**296**	**100.0%**	**3.99 [2.28, 5.70]**
Heterogeneity: Tau² = 3.53; Chi² = 21.60, df = 7 (P = 0.003); I² = 68%								
Test for overall effect: Z = 4.57 (P < 0.00001)								

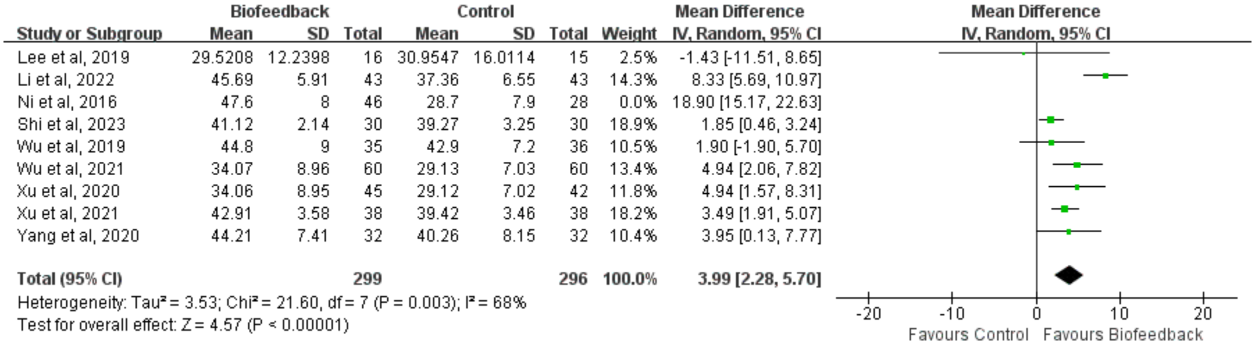

Figure S2 Sensitivity−analysis of the mean resting pressure of the anal canal

Appendix 1

检索条件	检索范围
（主题：位前切除综合征 + 前切除综合征 + 直肠癌术后 + 直肠癌低位保肛术 + Lars + Ars）AND（篇关摘：生物反馈(精确)）	资源范围：总库; 中英文扩展; 时间范围：发表时间：2012-01-01到2024-06-30;更新时间：不限;

Chinese National Knowledge Infrastructure (CNKI): 44

文献类型	检索式	检索结果
期刊、学位、会议...	(主题:(低位前切除综合征 or 前切除综合征 or 直肠癌术后 or 直肠癌低位保肛术 or Lars or Ars) and 题名或关键词:(生物反馈)) and 发表时间:2012-2024	61

Wan Fang Data: 61

检索结果	检索表达式
57	(((((摘要=低位前切除综合征 OR 摘要=前切除综合征) OR 摘要=直肠癌术后) OR 摘要=直肠癌低位保肛术) OR 摘要=Lars) OR 摘要=Ars) AND 摘要=生物反馈) AND (years:[2012 TO 2024])

VIP database: 57

检索表达式	结果
((低位前切除综合征 or 前切除综合征 or 直肠癌术后 or 直肠癌低位保肛术 or Lars or Ars) AND (生物反馈)) AND 2012-2024[日期]	44

China Biology Medicine disc (CBM): 44

Query	Results
Search: **((((Low anterior resection syndrome[MeSH Terms]) OR (Lars)) OR (anterior resection syndrome)) OR (resection for rectal cancer)) AND (Biofeedback)** Filters: **from 2012 - 2024**	36

Pubmed: 36

#1	(Low anterior resection syndrome):ti,ab,kw OR (Lars):ti,ab,kw OR (anterior resection syndrome):ti,ab,kw OR (resection for rectal cancer):ti,ab,kw	S▾	Limits	2290
#2	(Biofeedback):ti,ab,kw	S▾	Limits	4600
#3	#1 AND #2<br>with Publication Year from 2012 to 2024, in Trials		Limits	20

Cochrane Library: 20

Topic | Example: oil spill* mediterranean — (Low anterior resection syndrome) OR (Lars) OR (anterior resection syndrome) OR (resecti

And | Topic | Example: oil spill* mediterranean — Biofeedback

Publication Date | YYYY-MM-DD — 2012-01-01 to YYYY-MM-DD — 2024-06-30

Web of Science: 59

('low anterior resection syndrome'/exp OR 'low anterior resection syndrome' OR lars OR 'anterior resection syndrome'/exp OR 'anterior resection syndrome' OR 'resection for rectal cancer') AND biofeedback:ti,ab,kw AND [randomized controlled trial]/lim AND [2012-2024]/py 8

Embase: 8

Additional records identified through other sources (n = 3): Wang LQ et al,2020; Gai JJ et al,2015; Bai JB et al,2013
